# Scapular morphology does not predict supraspinatus tendon tear propagation following an individualised exercise therapy programme

**DOI:** 10.1002/jeo2.12072

**Published:** 2024-07-04

**Authors:** Ehab M. Nazzal, Luke T. Mattar, Philipp W. Winkler, Adam J. Popchak, James J. Irrgang, Albert Lin, Volker Musahl, Richard E. Debski

**Affiliations:** ^1^ Orthopaedic Robotics Laboratory Pittsburgh Pennsylvania USA; ^2^ Department of Orthopaedic Surgery University of Pittsburgh Medical Center Pittsburgh Pennsylvania USA; ^3^ Department of Bioengineering, Swanson School of Engineering University of Pittsburgh Pittsburgh Pennsylvania USA; ^4^ Department of Physical Therapy University of Pittsburgh Pittsburgh Pennsylvania USA; ^5^ Department of Orthopaedic Surgery University of Pittsburgh Pittsburgh Pennsylvania USA

**Keywords:** acromion morphology, critical shoulder angle, exercise therapy, rotator cuff tear propagation, supraspinatus tendon tear

## Abstract

**Purpose:**

To determine whether scapular morphology could predict isolated supraspinatus tendon tear propagation after exercise therapy. We hypothesised that a larger critical shoulder angle (CSA) and type III acromial morphology predict a positive change in tear size.

**Methods:**

Fifty‐nine individuals aged 40–70 years with isolated symptomatic high‐grade partial or full‐thickness supraspinatus tendon tears were included. Individuals participated in a structured, individualised 12‐week exercise therapy programme and underwent ultrasound to measure tear size at baseline and 12 months following therapy. Computed tomography images were segmented to create three‐dimensional subject‐specific bone models and reviewed by three trained clinicians to measure CSA and to determine acromion morphology based on the Bigliani classification. A binary logistic regression was performed to determine the predictive value of CSA and acromion morphology on tear propagation.

**Results:**

The CSA was 30.0 ± 5.4°. Thirty‐one individuals (52.5%) had type II acromial morphology, followed by type III and type I morphologies (25.4% and 22.0%, respectively); 81.4% experienced no change in tear size, four (6.8%) individuals experienced tear propagation and seven (11.9%) individuals had a negative change in tear size. No significant difference in tear propagation rates based on CSA or acromion morphology (not significant [NS]) was observed. The model predicted tear size status in 81.4% of cases but only predicted tear propagation 8.3% of the time. Overall, CSA and acromion morphology only predicted 24.3% (*R* ^2^ = 0.243) of variance in tear propagation (NS).

**Conclusions:**

CSA and acromion morphology were NS predictors of tear propagation of the supraspinatus tendon 12 months following an individualised exercise therapy programme.

**Level of Evidence:**

II.

**Abbreviation**
3D: Three dimensional
AP: anterior‐posterior
BMI: body mass index
CSA: critical shoulder angle
CT: computed tomography
ICC: intraclass correlation coefficients
IRB: institutional review board
NS: not significant
ROM: range of motion

## INTRODUCTION

Rotator cuff injuries are one of the most common adult musculoskeletal complaints [26]. Damage to the supraspinatus tendon is the most common, with age‐related biomechanical and histological changes to the supraspinatus tendon resulting in susceptibility to degeneration and ultimately rupture [4, 7]. Given the socioeconomic impact of rotator cuff disease, defining anatomic risk factors associated with rotator cuff tendon tears, specifically bony morphology of the glenohumeral joint, is increasingly important. Two commonly studied morphologies are critical shoulder angle (CSA) and acromion morphology. The CSA is a combination of glenoid inclination angle and acromion index [22], while acromion morphology characterises acromion shape as a risk factor for mechanical impingement [3]. The role of CSA on degenerative rotator cuff disease has been extensively investigated in clinical studies [10, 24, 28], with 35° being the established threshold for risk of degenerative rotator cuff disease [22]. Biomechanically, CSA is thought to alter the resultant force of the deltoid vector during arm abduction, inducing a compensatory load on the supraspinatus to maintain joint stability and arm stabilisation [9, 34]. Additionally, increases in glenoid inclination can increase the loading of the supraspinatus and predispose the tendon to degenerative changes [23]. For acromion morphology and rotator cuff tendon tears, the increased slope is thought to decrease the volume of the subacromial space, increasing bony contact with the supraspinatus tendon and increasing the risk of degenerative damage [3, 14].

Treatment of rotator cuff tendon tears consists of conservative therapy and surgical management. Exercise therapy is generally an effective conservative treatment with favourable outcomes for partial‐thickness tears, degenerative nontraumatic full‐thickness tears and massive tears that cannot be repaired surgically [8, 13]. However, given the risk of rotator cuff repair failure in large or massive tears, which has been noted to be as high as 94% in various case series [1], defining changes in tear size following exercise therapy as a treatment for degenerative rotator cuff tendon tears is especially important. Exercise therapy strengthens the rotator cuff and scapular musculature [31] and facilitates a normal glenohumeral joint contact path length [2], increasing joint stability by balancing the muscle forces at the glenohumeral joint and preventing further tear propagation. Identifying which scapular morphologies may fare worse with exercise therapy is important for surgeons and physical therapists to understand the development of a nonoperative treatment regimen and may help in the creation of individualised exercise therapy programmes that maximise function and satisfaction.

The objective of this study was to determine whether scapular morphology, specifically CSA and acromion morphology, could predict tear size propagation after exercise therapy in individuals with isolated supraspinatus tendon tears. We hypothesised that a larger CSA and hooked acromion morphology predict supraspinatus tendon tear propagation at one year from baseline measurements following a 12‐week individualised exercise therapy programme.

## METHODS

Fifty‐nine individuals (age 58.8 ± 8.8 years, body mass index 28.2 ± 5.1 kg/m^2^, 31 males [52.5%] and 28 females [47.5%]) were recruited to participate in this study and provided Institutional Review Board‐approved written informed consent prior to performance of any research procedure. Individuals were included if they had: (1) atraumatic high‐grade partial (articular‐sided, intratendinous) or full‐thickness rotator cuff tears isolated to the supraspinatus tendon, (2) were above the age of 40 years and (3) had at least 110° of humerothoracic elevation. Individuals were excluded if they had: (1) a diagnosis of glenohumeral osteoarthritis in the affected shoulder, (2) a work‐related injury, (3) asymptomatic rotator cuff tear, (4) severe capsular tightness as evidenced by less than 30° of internal or external rotation, (5) diabetes mellitus and (6) duration of shoulder pain exceeding 12 months. All individuals underwent a 12‐week (12 visits in total) structured and individualised exercise therapy programme administered by a physical therapist. The exercise therapy programme utilised individualised criteria related to each individual's pain, range of motion (ROM) and strength to tailor the interventions to each individual's clinical presentation [12]. Exercises prescribed throughout the programme targeted passive ROM, active assistive ROM, activities of daily living and strengthening of key muscle groups [21]. The programme consisted of two visits per week for 4 weeks, then one visit per week for a total of 2 weeks and finally one visit bi‐weekly until the conclusion of the 12 weeks. The programme also included a home exercise programme and visit frequency was constructed as such to simulate current clinical practice and provide opportunities to progress while maximising compliance with the home exercise programme prescribed. The overarching framework focused on restoring passive glenohumeral ROM and strengthening the rotator cuff and scapular muscles. However, depending on the initial staging of the individual based on tissue irritability, the impairments that needed to be addressed and response to treatment, the selection and progression of interventions were individualised.

Ultrasound was used to quantify the anterior‐posterior (AP) tear size within the supraspinatus tendon. These measurements were made by a musculoskeletal radiologist. Ultrasound has been shown to be sensitive and reliable for the detection and quantification of rotator cuff tears with an accuracy of 1.0 mm or less [25, 33, 35]. Supraspinatus tear size was quantified as the AP distance of the tear measured perpendicular to a line tangent to the posterior edge of the long head of the biceps tendon. Measurements of tear size were performed at baseline (prior to initiation of exercise therapy programme) and at 12‐month follow‐up. Changes in AP tear size (12‐month tear size—baseline tear size) greater than 5 mm were considered clinically meaningful [16]. Thus, any individual whose tear changed by at least 5 mm was considered to have a change in tear size, while those who did not change by at least 5 mm were considered to have no change in tear size. Tear size thickness was classified as either less than or greater than 50% and tear size thickness was not a criterion for tendon tear propagation.

All individuals underwent a computed tomography (CT) scan of the involved shoulder prior to initiation of the exercise therapy programme. Individual‐specific three‐dimensional (3D) models of the scapula were then created from the CT scans. The slice thickness was 0.625 mm and the approximate pixel size was 0.471 × 0.471 [18]. CT images were segmented using Mimics 20 (Materialise) to create individual‐specific bone models of the scapula.

**Figure 1:**
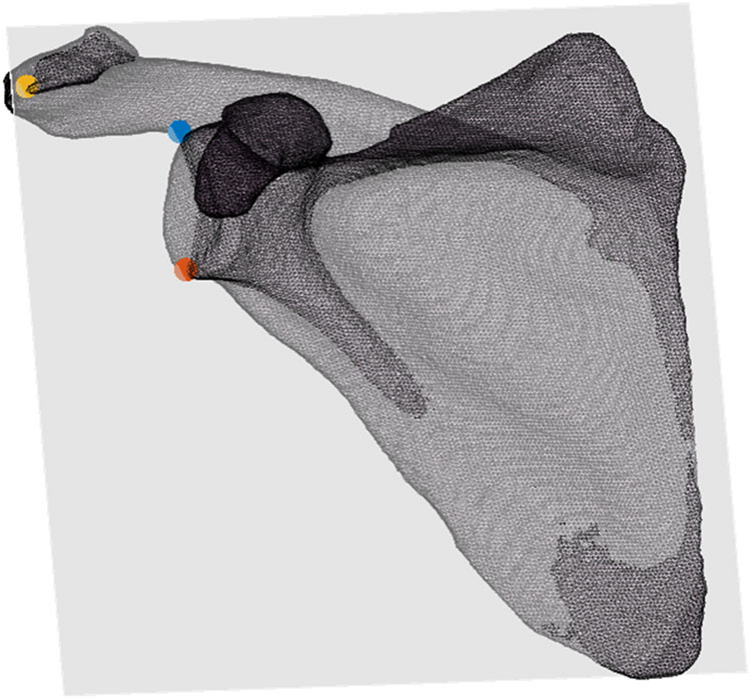
Illustration of determination of anatomic landmarks projected onto the scapular plane viewed in the coronal plane. Red, inferior glenoid; blue, superior glenoid; orange, lateral acromion process.

**Figure 2:**
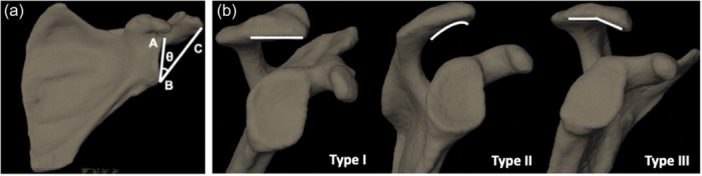
(a) Example of a bone model segmented from an individual's computed tomography scan, illustrating the critical shoulder angle defined as the angle (*θ*), between the line joining the superior point of the glenoid (A) and inferior glenoid (B) and the line joining the lateral edge of the acromion (C) to the inferior edge of the glenoid (B). (b) Illustration of three different acromion morphologies defined by the Bigliani Classification [15]. From left to right, Type I (flat), Type II (curved) and Type III (hooked).

Utilising the bone models, CSA was determined for all individuals. CSA was quantified using a custom‐written Matlab code (2020a, MathWorks) and each scapula was oriented on the *z* axis (superior‐inferior glenoid vector) and *x* axis (AP vector). The result was a position that mimicked a clinical X‐ray. Then, the most inferior and superior points on the glenoid rim and most lateral points on the acromion process were identified and projected onto the scapular plane (Figure 1). A vector was made from the inferior to the superior point on the glenoid pointing superiorly, and a second vector was determined from the inferior point of the glenoid rim to the most lateral point on the acromion pointing superiorly and laterally towards the acromion. The angle between these two vectors was calculated using the dot product and represented the CSA [6, 22] (Figure 2a). Blinded and repeated measures of CSA were also performed by three trained clinicians. Each clinician identified the points previously described on 10 scapulae, three times, with 1 week between each session. Intraclass correlation coefficients (ICCs) were determined to evaluate the intra‐ and inter‐rater reliability of the CSA measurements.

Acromion morphology based on the Bigliani classification [15] was also determined for all bone models (Figure 2b). Good–excellent intraobserver reliability was found when determining acromion morphology using radiographs previously [27, 32]. Therefore, a single trained clinician viewed the scapulae models once they had been oriented in the position mimicking a clinical X‐ray. The clinician then defined the acromion morphology as either a type I (flat), type II (curved) or type III (hooked).

Outcome parameters included the CSA, acromion type and AP tear size. Tear size was collected at both baseline and 12 months, whereas CSA and acromion type were determined only at baseline. Three different categories were created based on the classification of changes in tear size, including no change (between −5 and 5 mm), increased tear size (≥5 mm) and negative change in tear size (≤5 mm). These categories were then used to analyse patients based on CSA thresholds and acromion morphology. A binary logistic regression analysis was performed to determine the predictive value of CSA and acromion morphology type on tear propagation. CSA was a continuous variable and acromion type was coded as 1, 2 and 3 for types I, II and III, respectively. Because the interest was in tear size progression and not negative change in tear size, tear propagation was coded as a dichotomous variable in which 0 corresponded to ‘no change in tear size’ and 1 corresponded to ‘change in tear size’. Based on previously published literature on the correlation between CSA and rotator cuff tendon tear propagation [6], an a priori sample size analysis was performed (G*Power Version 3.1; Heinrich‐Heine‐Universität Düsseldorf), which indicated that 57 individuals would be required to achieve 80% power (*α* = 0.05). For all statistical tests, the level of significance was set at *p* < 0.05.

## RESULTS

Analysis of the 3D models of the scapulae yielded measurements of scapular morphology with high ICCs. Excellent intra‐rater [ICC, 0.99 95% confidence interval (0.95,0.99)] and inter‐rater [(ICC, 0.99 95% confidence interval (0.94, 0.99)] reliability of measurements were found from the blinded and repeated measurements of CSA. The 59 included individuals were 58.8 ± 6.8 years of age and had a CSA of 30.0 ± 5.4°. Individuals were also categorised based on acromion morphology. Most individuals had a type II acromion morphology (52.5%), followed by type III (25.4%) and type I (22.0%).

**Figure 3:**
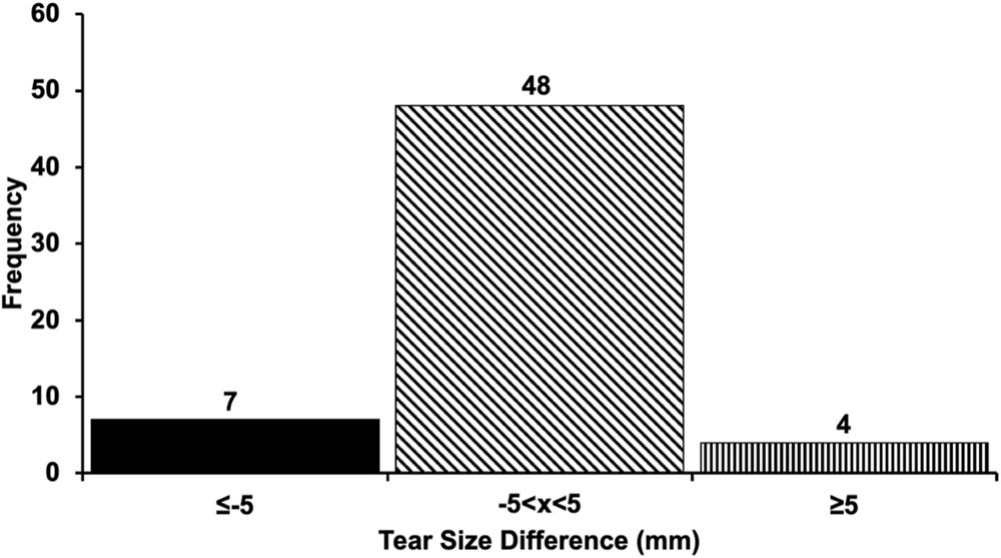
Histogram demonstrating the number of individuals who had no anterior‐posterior tear propagation (slanted line fill), positive change in tear size (straight line fill) and negative change in tear size (solid black fill). Detectable change was defined as an absolute change in tear size ≥5.0 mm.

Most individuals did not have any changes in tear size at the end of the study period. Thirty (50.8%) individuals had tears in their dominant side and 29 (49.2%) individuals in their nondominant side. Eighteen and 41 individuals had high‐grade partial and full‐thickness tears, respectively. Table 1 summarises the demographic data of the study's cohort. At baseline, the tear size was 12.6 ± 5.8 mm and ranged from 4.4 to 28.3 mm. Twenty‐four individuals had tears less than or equal to 10.0 mm and 35 individuals had tears greater than 10.0 mm and less than 30.0 mm. None of the individuals underwent surgery during the follow‐up period. Following exercise therapy, 48 individuals (81.4%) had no change in tear size when comparing the difference between baseline and 12‐month tear size measurements, seven individuals (11.9%) had a negative change in tear size and four individuals (6.8%) had a positive change in tear size representing supraspinatus tendon tear propagation (Figure 3). For the four individuals that experienced tear propagation, increases of 8.0 ± 3.1 mm were observed (range: 5.2–12.4 mm).

**Table 1:** Baseline demographics for individuals with symptomatic isolated supraspinatus tears.

	*n*	%
Type of work		
Mostly sedentary	26	44.0
Sedentary, substantial walking	3	5.1
Moderately active, some lifting	26	44.1
Demanding	4	6.8
Race		
White/Caucasian	52	88.1
Black or African American	5	8.5
American Indian or Alaska Native	1	1.7
Asian	1	1.7
Native Hawaiian or Other Pacific Islander	0	0.0
Other	0	0.0
Duration of shoulder pain (month)	8	13.6
≤1	15	25.4
>1– ≤3	17	28.8
>3– <6	19	32.2
≥6		
Smoking status		
Smoke currently	3	5.1
Smoked in past	20	33.9

**Figure 4:**
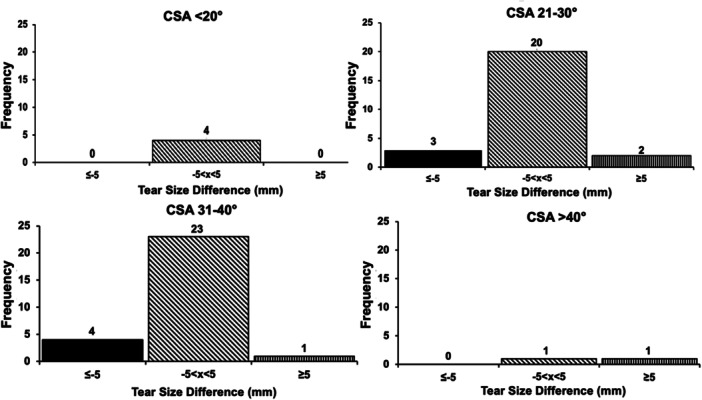
Histograms demonstrating distribution of no anterior‐posterior tear propagation (slanted line fill), positive change in tear size (straight line fill) and negative change in tear size (solid black fill) based on critical shoulder angle (CSA). Detectable change was defined as an absolute change in tear size ≥5.0 mm.

**Figure 5:**
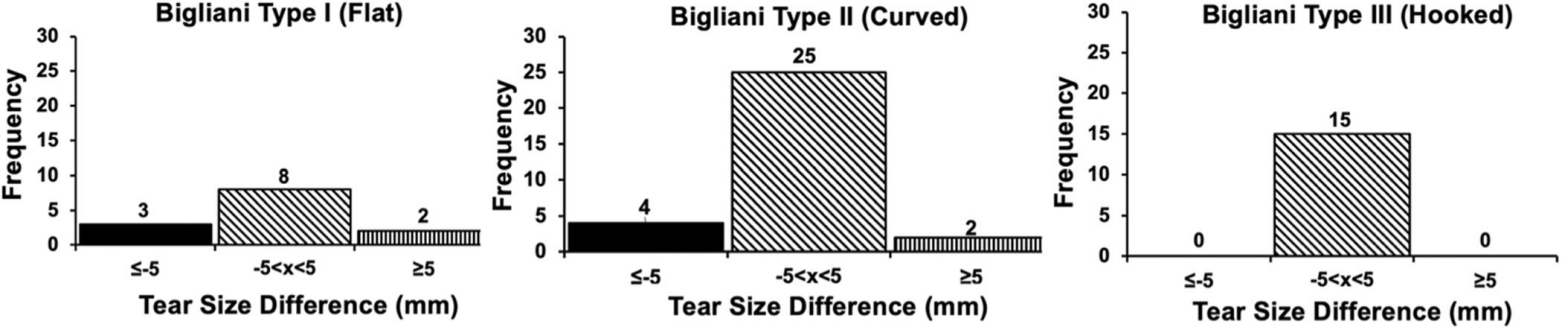
Histograms demonstrating distribution of no anterior‐posterior tear propagation (slanted line fill), positive change in tear size (straight line fill) and negative change in tear size (solid black fill) based on acromion morphology (Bigliani classification [15]). Detectable change was defined as an absolute change in tear size ≥5.0 mm.

No observed relationship could be found between CSA, acromion morphology and tear size propagation, regardless of tear size threshold. In each CSA threshold, most individuals did not have any change in tear size (Figure 4). Additionally, regardless of acromion morphology, most individuals did not experience any tear propagation or negative change in tear size (Figure 5). No significant differences were identified in the proportion of patients who experienced tear propagation between different CSA thresholds (not significant [NS]) and between the different acromion morphology types (NS).

The ability of the binary logistic regression model using scapular morphology to predict tear size status varied. Tear size status was correctly predicted in 81.4% of cases. The model was more accurately able to predict no change in tear status, doing so in 100.0% of cases (47/47). Change in tear size was not predicted as accurately, as the model only correctly predicted this in one of 11 (8.3%) cases (Table 2). Altogether, CSA and Bigliani type were only able to predict 24.3% (*R* ^2^ = 0.243) of variance in tear propagation.

**Table 2:** Predictive model outcomes, utilising binary logistic regression with critical shoulder angle and acromion morphology type as input and tear size as output.

Parameter	Rate of correct prediction (%)
Tear size status (no change, negative or propagation)	81.4
No change in tear size	100.0
Tear propagation	8.3

## DISCUSSION

The most important finding of this study is that while the logistic regression model successfully predicted individuals experiencing no change in tear size, it could not predict changes in tear size based on acromion type and CSA. Additionally, contrary to our hypothesis, CSA threshold and acromion type were NS predictors of tear propagation in individuals with isolated supraspinatus tendon tears following an exercise therapy programme.

Tear size propagation is an important metric for evaluating nonoperative management of rotator cuff tendon tears. The rate of tear size enlargement over time varies, ranging from 39% to 52% increases in various observational studies [17, 30, 36]. While compelling for early surgical intervention, these previously reported rates of tear propagation are likely inflated due to a variety of factors, including inconsistent comparison of asymptomatic versus symptomatic tears, extended follow‐up periods that are more likely to capture increases in tear size and utilisation of lower thresholds of tear propagation, resulting in potentially clinically insignificant definitions of tear propagation. Because the present study utilised a standardised, repeatable method of measuring CSA and predefined acromion morphology that eliminated these potential confounders, the observed 7% rate of tear propagation in symptomatic patients with clinically significant tear size propagation is likely more accurate compared to findings of previous studies [6, 17, 30, 36]. Additionally, although the present study's 12‐month follow‐up period may be shorter than those of previous studies, failure of nonoperative treatment has been shown to occur within the first 12 weeks of initiation [13], making our 12‐month follow‐up period an appropriate timepoint to track tear propagation.

The present study did not find any CSA threshold and acromion morphology to be predictive of supraspinatus tendon tear propagation, even after exercise therapy. Moreover, the developed model only predicted positive changes in tear size in 8.3% of cases. The findings of the present investigation are similar to a recent retrospective analysis of the relationship between bony morphology and rotator cuff tendon tears, which found no correlation between CSA magnitude and tear size progression [6]. Additionally, hooked acromion morphology was NS associated with tear propagation. While the present study's analysis of Bigliani classification as a predictor of tear propagation is novel, acromion morphology has been extensively studied regarding rotator cuff tear and retear after repair. 3D magnetic resonance imaging was recently utilised to evaluate the relationship between hooked acromia, rotator cuff tears and healing. Patients with rotator cuff tendon tears were found to more often have upsloping acromia (vs. the traditional down‐sloping, hooked acromial morphology) refuting subacromial impingement as a driver of degenerative rotator cuff tendon tears [5]. The findings of this study support conclusions made previously, suggesting that the volume under the acromia, even in hooked morphologies, is not a predictor of tear propagation [38].

Finally, the role of physical activity on tear propagation must be considered. In this investigation, all patients underwent exercise therapy, which included periods of increased utilisation of their injured shoulder. Even with this increased activity, 93.2% of patients within the present cohort did not experience a positive change in tear size. While hand dominance has been treated as a proxy for increased upper extremity functional demand, the results on its association with the progression of rotator cuff tendon disease are inconsistent [19, 37]. Other metrics, including occupational demand and shoulder activity level, have also been assessed and no clear relationship between these factors and the risk of tear progression has been identified [11]. Meanwhile, exercise therapy for rotator cuff tendon tears has been shown to improve pain relief, function, ROM and glenohumeral/scapulothoracic kinematics [2]. The present study informs clinicians that even for those with CSA values and acromion morphologies that traditionally predispose to the presence of rotator cuff tendon tears, the risk of tear propagation does not increase, even after a focused, intensive exercise therapy programme. These findings are important to surgeons and therapists because they suggest that regardless of scapular morphology, patients will not increase their risk of tear propagation with exercise therapy. Additionally, the low predictive value of larger CSA and hooked acromia on tear propagation suggest that these parameters, while certainly important to consider, do not serve as indications for earlier operative intervention.

The findings of this investigation should be interpreted with consideration of our methodology and data collection. As previously mentioned, the radiographic parameters were measured based on individual bone models developed from CT scans that allowed for standardised scapular planar orientation and precise determination of anatomic landmarks to maximise the accuracy of measurements that are not usually available in the clinical setting.

The current study has a few limitations worth noting. First, although there were enough individuals included in our sample to reach adequate power, the low number of subjects with tear propagation limits the variation in data, thereby decreasing the predictive value of the model. Additional limitations include the absence of a control group and the scope of results, which solely delineate occurrences up to the 12‐month follow‐up period. Further, due to the low number of individuals that experienced tear propagation, both high‐grade partial and full‐thickness groups had to be analysed in one model. Irrespective of this, the small number of individuals with tear propagation, in combination with the agreement of our results with similar literature [6], strengthens the implications that CSA and acromion morphology do not need to be factors that preclude exercise therapy to prevent tear propagation. Future studies will consider separating the components of the CSA into glenoid inclination angle and lateral acromion edge length to determine their individual effects on tear propagation. Additionally, further investigations on the relationship between exercise therapy and both patient‐reported outcomes and joint kinematics within this cohort should be considered to further elucidate the clinical significance of tear size propagation.

## CONCLUSIONS

The present investigation is a novel study utilising two radiographic parameters commonly implicated in the pathogenesis of degenerative rotator cuff tendon tears. The results demonstrate that CSA and acromion morphology were NS predictors of tear propagation of the supraspinatus tendon 12 months following an individualised exercise therapy programme. These findings contribute to our understanding of rotator cuff tendon tear propagation [20, 29] and its relationship to scapular morphology [6] and suggest that elevated CSA and hooked acromion morphology should not be considered potential reasons to delay exercise therapy, especially given the benefits of therapy including pain relief, restoration of ROM and improvements in joint kinematics.

## AUTHOR CONTRIBUTIONS

Ehab M. Nazzal, Luke T. Mattar, Philipp W. Winkler, Adam J. Popchak, James J. Irrgang, Albert Lin and Volker Musahl made substantial contributions to the conception or design of the work; or the acquisition, analysis or interpretation of data or the creation of new software used in the work and drafted the work or revised it critically for important intellectual content. Richard E. Debski made substantial contributions to the conception or design of the work; or the acquisition, analysis or interpretation of data or the creation of new software used in the work; drafted the work or revised it critically for important intellectual content; approved the version to be published and agree to be accountable for all aspects of the work in ensuring that questions related to the accuracy or integrity of any part of the work are appropriately investigated and resolved.

## CONFLICT OF INTEREST STATEMENT

The authors declare no conflict of interest.

## ETHICS STATEMENT

Ethics approval was obtained through the Institutional Review Board (IRB), provided by the University of Pittsburgh (no. STUDY19020103). All participants consented prior to participation.

## Data Availability

All data generated and/or analysed during this study are included in this published article [and its Supplementary Information files].
